# Arcs and Stages: Retrospectively Conceptualizing a Gerontological Career

**DOI:** 10.1093/geroni/igaa057.2328

**Published:** 2020-12-16

**Authors:** Kenneth Hepburn

**Affiliations:** Emory University, Atlanta, Georgia, United States

## Abstract

The arc of a gerontologist’s career is that of a “work in progress,” work unlikely to be completed. Early efforts might develop interdisciplinary collaborations and establish the principles and mechanisms of a central line of work and inquiry. Mid-stage work may entail expansion and adaptation of preliminary efforts and identification of exciting areas of exploration that both fit within the gerontologist’s overall thematic trajectory and extend beyond the reasonable scope of pursuit. In late stage, the most pressing concern is to sustain, but not constrain, the work’s trajectory. This may best be accomplished by identifying and supporting students and early career researchers who are passionate about the work and who are likely to move it forward and expand it in their own unique and divergent ways. Emeriti gerontologists may seek to remain generatively engaged in ways that both contribute to and let go of the continuing arc of the work.

